# *fac*-Bromidotricarbonyl[2-(diisopropylphosphanyl)benzaldehyde-κ^2^ *O*,*P*]rhenium(I)

**DOI:** 10.1107/S1600536812035957

**Published:** 2012-08-23

**Authors:** Christos Apostolidis, Martin Ahlmann, Olaf Walter

**Affiliations:** a European Commission, Joint Research Centre, Institute for Transuranium Elements, Hermann-von-Helmholtz-Platz 1, 76344 Eggenstein-Leopoldshafen, Germany; b IKFT, KIT-Campus Nord, Hermann-von-Helmholtz-Platz 1, 76344 Eggenstein-Leopoldshafen, Germany

## Abstract

The structure of the title complex, [ReBr(C_13_H_19_OP)(CO)_3_], displays a facial coordination of the three CO ligands and a κ^2^ *O*,*P* coordination mode of the 2-diisopropyl­phosphino­benzaldehyde ligands. The Re—C bond distance for the CO ligand *trans* to the P atom is, due to its *trans* influence, elongated to 1.943 (3) Å, showing that this CO ligand is more weakly bound to the Re centre than the other two.

## Related literature

For the structures of halo-*fac*-tricarbonyl-[κ^2^ *O*,*P*-(ligand)]rhenium(I) complexes with ligands based on 2-diphenyl­phosphinobenzaldehyde or 2-diphenyl­phosphinobenzoic acid derivatives, see: Correia *et al.* (2001 ▶); Chen *et al.* (2001 ▶); Palma *et al.* (2004 ▶).
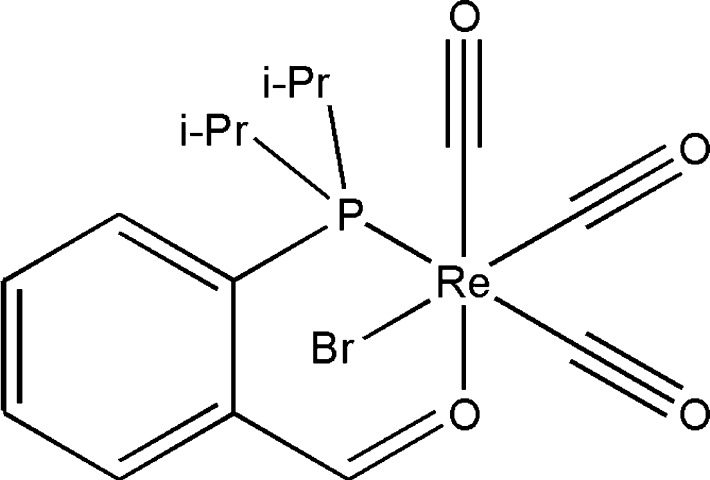


## Experimental

### 

#### Crystal data

[ReBr(C_13_H_19_OP)(CO)_3_]
*M* *_r_* = 572.39
Monoclinic, 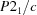

*a* = 10.750 (2) Å
*b* = 15.194 (3) Å
*c* = 13.699 (3) Å
β = 125.29 (3)°
*V* = 1826.4 (9) Å^3^
*Z* = 4
Mo *K*α radiation
μ = 8.94 mm^−1^
*T* = 200 K
0.43 × 0.33 × 0.31 mm

#### Data collection

Siemens SMART 1000 CCD diffractometer
Absorption correction: numerical *SADABS* (Bruker, 1997 ▶) *T*_min_ = 0.631, *T*_max_ = 1
18487 measured reflections
4436 independent reflections
4095 reflections with *I* > 2σ(*I*)
*R*_int_ = 0.027

#### Refinement

*R*[*F* ^2^ > 2σ(*F* ^2^)] = 0.018
*wR*(*F* ^2^) = 0.045
*S* = 1.11
4436 reflections
221 parameters
H atoms treated by a mixture of independent and constrained refinement
Δρ_max_ = 0.51 e Å^−3^
Δρ_min_ = −1.04 e Å^−3^

### 

Data collection: *SMART* (Bruker, 1997 ▶); cell refinement: *SAINT* (Bruker, 1997 ▶); data reduction: *SAINT*; program(s) used to solve structure: *SHELXS97* (Sheldrick, 2008 ▶); program(s) used to refine structure: *SHELXL97* (Sheldrick, 2008 ▶); molecular graphics: *XPMA* (Zsolnai, 1996 ▶), *ORTEP* (Farrugia, 1997 ▶); software used to prepare material for publication: *publCIF* (Westrip, 2010 ▶).

## Supplementary Material

Crystal structure: contains datablock(s) I, global. DOI: 10.1107/S1600536812035957/vn2046sup1.cif

Structure factors: contains datablock(s) I. DOI: 10.1107/S1600536812035957/vn2046Isup2.hkl

Supplementary material file. DOI: 10.1107/S1600536812035957/vn2046Isup3.mol

Additional supplementary materials:  crystallographic information; 3D view; checkCIF report

## Figures and Tables

**Table 1:** Selected bond lengths (Å)

Re1—C14	1.901 (3)
Re1—C15	1.943 (3)
Re1—C16	1.915 (3)
Re1—O1	2.1739 (18)
Re1—P1	2.4655 (13)
Re1—Br1	2.6116 (6)

**Fig. 1.**
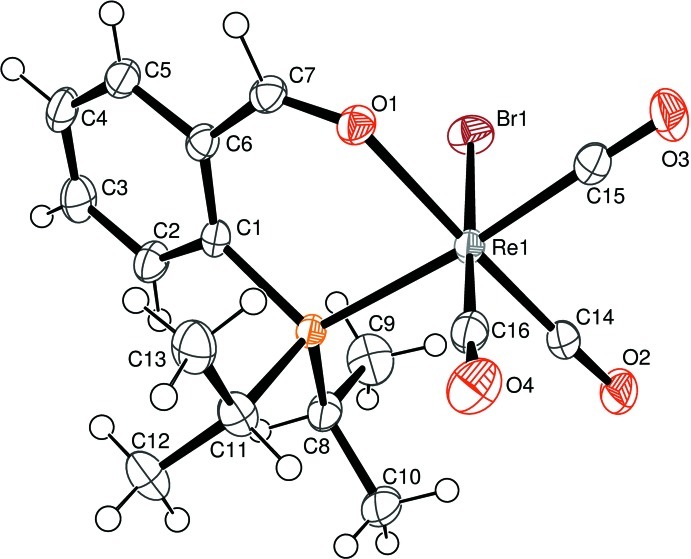
Molecular structure of the title compound with displacement ellipsoids at the 50% probability level.

## supplementary crystallographic information

### Experimental

The title compound was obtained from the reaction of 371.0 mg (1.38 mmol) 2-diisopropylphosphinobenzaldehydedimethylacetale and 561.5 mg (1.38 mmol) bromo-pentacarbonyl-rhenium(I) in refluxing thf (30 ml) after recrystallization from diethylether in 93% yield (736 mg). Single crystals of the title compound were grown in an NMR tube with CDCl_3_ as the solvant.

### Refinement

The position of the H atom located at the aldehyde carbon atom was localized and refined together with its isotropic displacement parameter. The positions of all other H atoms were calculated at geometrical positions according to the hybridization of the atoms they are bound to. The isotropic *U* values of the hydrogen atoms were refined group-wisely.

### Crystal data

**Table:**

[ReBr(C_13_H_19_OP)(CO)_3_]	*F*(000) = 1088
*M**_r_* = 572.39	*D*_x_ = 2.082 Mg m^−^^3^
Monoclinic, *P*2_1_/*c*	Mo *K*α radiation, λ = 0.71073 Å
Hall symbol: -P 2ybc	Cell parameters from 165 reflections
*a* = 10.750 (2) Å	θ = 2.3–28.3°
*b* = 15.194 (3) Å	µ = 8.94 mm^−^^1^
*c* = 13.699 (3) Å	*T* = 200 K
β = 125.29 (3)°	Cube, orange
*V* = 1826.4 (9) Å^3^	0.43 × 0.33 × 0.31 mm
*Z* = 4	

### Data collection

**Table:**

Siemens SMART 1000 CCD diffractometer	4436 independent reflections
Radiation source: fine-focus sealed tube	4095 reflections with *I* > 2σ(*I*)
Graphite monochromator	*R*_int_ = 0.027
Detector resolution: 8 pixels mm^-1^	θ_max_ = 28.3°, θ_min_ = 2.3°
ω scans	*h* = −14→14
Absorption correction: numerical *SADABS* (Bruker, 1997)	*k* = −19→19
*T*_min_ = 0.631, *T*_max_ = 1	*l* = −17→17
18487 measured reflections	

### Refinement

**Table:**

Refinement on *F*^2^	Primary atom site location: structure-invariant direct methods
Least-squares matrix: full	Secondary atom site location: difference Fourier map
*R*[*F*^2^ > 2σ(*F*^2^)] = 0.018	Hydrogen site location: inferred from neighbouring sites
*wR*(*F*^2^) = 0.045	H atoms treated by a mixture of independent and constrained refinement
*S* = 1.11	*w* = 1/[σ^2^(*F*_o_^2^) + (0.0228*P*)^2^ + 0.987*P*] where *P* = (*F*_o_^2^ + 2*F*_c_^2^)/3
4436 reflections	(Δ/σ)_max_ = 0.002
221 parameters	Δρ_max_ = 0.51 e Å^−^^3^
0 restraints	Δρ_min_ = −1.04 e Å^−^^3^

### Special details

**Table:**

Experimental. Spectroscopic data: ^1^H{^31^P} NMR (CDCl_3_): δ = 9.71, s, 1H, CHO; 7.99, dd, ^3^J_HH_ = 7.5 Hz, ^4^J_HH_ = 1.2 Hz, 1H, CH(arom); 7.89, dt, ^3^J_HH_ = 7.5 Hz, ^4^J_HH_ = 1.2 Hz, 1H, CH(arom); 7.78, dt, ^3^J_HH_ = 7.5 Hz, ^4^J_HH_ = 1.2 Hz, 1H, CH(arom); 7.72, dd, ^3^J_HH_ = 7.5 Hz, ^4^J_HH_ = 1.2 Hz, 1H, CH(arom); 2.94, hept, ^3^J_HH_ = 7.0 Hz, 1H, CH(Me)_2_; 2.55, hept, ^3^J_HH_ = 6.9 Hz, 1H, CH(Me)_2_; 1.38, d, ^3^J_HH_ = 7.0 Hz, 3H, CH_3_; 1.31, d, ^3^J_HH_ = 7.0 Hz, 3H, CH_3_; 1.03, d, ^3^J_HH_ = 7.0 Hz, 3H, CH_3_; 0.97, d, ^3^J_HH_ = 6.9 Hz, 3H, CH_3_; ^31^P{^1^H} NMR (CDCl_3_): δ = 19.7, s; ^13^C{^31^P}{^1^H} NMR (CDCl_3_): δ = 200.6; 196.2; 190.3; 141.0; 136.0; 132.5; 131.6; 27.9; 23.3; 18.9; 18.2; 17.2; 16.2; IR ν_CO_ [cm^-1^]: 2041, 1949, 1905, 1634; UV vis (CHCl_3_): 414 nm (ε 2142); 261 nm (ε 9780); UV vis (MeCN): 391 nm (ε 1794); 312 nm (sh); 257 nm (ε 7952); 214 nm (ε 27000)
Geometry. All e.s.d.'s (except the e.s.d. in the dihedral angle between two l.s. planes) are estimated using the full covariance matrix. The cell e.s.d.'s are taken into account individually in the estimation of e.s.d.'s in distances, angles and torsion angles; correlations between e.s.d.'s in cell parameters are only used when they are defined by crystal symmetry. An approximate (isotropic) treatment of cell e.s.d.'s is used for estimating e.s.d.'s involving l.s. planes.
Refinement. Refinement of *F*^2^ against ALL reflections. The weighted *R*-factor *wR* and goodness of fit *S* are based on *F*^2^, conventional *R*-factors *R* are based on *F*, with *F* set to zero for negative *F*^2^. The threshold expression of *F*^2^ > σ(*F*^2^) is used only for calculating *R*-factors(gt) *etc*. and is not relevant to the choice of reflections for refinement. *R*-factors based on *F*^2^ are statistically about twice as large as those based on *F*, and *R*-factors based on ALL data will be even larger. The data of the structure have been deposited at the CCDC with the reference number 894003 (Allen, 2002).

### Fractional atomic coordinates and isotropic or equivalent isotropic displacement parameters (Å^2^)

**Table:**

	*x*	*y*	*z*	*U*_iso_*/*U*_eq_	
Re1	0.096757 (10)	0.651316 (6)	0.822326 (9)	0.01984 (4)	
Br1	0.09257 (4)	0.70831 (2)	1.00026 (3)	0.03331 (7)	
P1	−0.17759 (7)	0.68270 (4)	0.68063 (6)	0.01901 (13)	
O1	0.0136 (2)	0.53062 (12)	0.85118 (17)	0.0246 (4)	
O2	0.2119 (2)	0.83034 (14)	0.8052 (2)	0.0381 (5)	
O3	0.4316 (2)	0.59455 (16)	1.0029 (2)	0.0446 (6)	
O4	0.1170 (3)	0.57547 (15)	0.6249 (2)	0.0387 (5)	
C1	−0.2889 (3)	0.63407 (17)	0.7300 (2)	0.0217 (5)	
C2	−0.4274 (3)	0.6705 (2)	0.6956 (3)	0.0294 (6)	
H2	−0.4596	0.7228	0.6525	0.035 (4)*	
C3	−0.5192 (3)	0.6307 (2)	0.7243 (3)	0.0343 (7)	
H3	−0.6104	0.6571	0.7013	0.035 (4)*	
C4	−0.4759 (3)	0.5528 (2)	0.7861 (3)	0.0320 (6)	
H4	−0.5383	0.5258	0.8038	0.035 (4)*	
C5	−0.3387 (3)	0.51476 (19)	0.8221 (2)	0.0275 (6)	
H5	−0.3092	0.4618	0.8638	0.035 (4)*	
C6	−0.2436 (3)	0.55548 (17)	0.7962 (2)	0.0220 (5)	
C7	−0.1008 (3)	0.50735 (17)	0.8444 (2)	0.0231 (5)	
C8	−0.2409 (3)	0.79816 (17)	0.6455 (3)	0.0273 (6)	
H8	−0.3526	0.7981	0.5939	0.038 (9)*	
C9	−0.1909 (4)	0.8520 (2)	0.7555 (3)	0.0427 (8)	
H9A	−0.0820	0.8576	0.8046	0.049 (4)*	
H9B	−0.2227	0.8231	0.8000	0.049 (4)*	
H9C	−0.2365	0.9093	0.7316	0.049 (4)*	
C10	−0.1865 (4)	0.8395 (2)	0.5746 (3)	0.0390 (8)	
H10A	−0.2189	0.8998	0.5572	0.049 (4)*	
H10B	−0.2292	0.8077	0.5011	0.049 (4)*	
H10C	−0.0773	0.8368	0.6209	0.049 (4)*	
C11	−0.2622 (3)	0.6290 (2)	0.5325 (3)	0.0288 (6)	
H11	−0.2026	0.6485	0.5029	0.030 (9)*	
C12	−0.4282 (4)	0.6553 (2)	0.4382 (3)	0.0429 (8)	
H12A	−0.4655	0.6244	0.3650	0.048 (4)*	
H12B	−0.4338	0.7176	0.4242	0.048 (4)*	
H12C	−0.4892	0.6404	0.4663	0.048 (4)*	
C13	−0.2484 (4)	0.5296 (2)	0.5436 (3)	0.0357 (7)	
H13A	−0.3162	0.5073	0.5620	0.048 (4)*	
H13B	−0.1455	0.5138	0.6063	0.048 (4)*	
H13C	−0.2749	0.5047	0.4694	0.048 (4)*	
C14	0.1677 (3)	0.76221 (19)	0.8091 (2)	0.0262 (6)	
C15	0.3070 (3)	0.6152 (2)	0.9370 (3)	0.0296 (6)	
C16	0.1055 (3)	0.60419 (18)	0.6968 (3)	0.0256 (6)	
H7	−0.101 (3)	0.4472 (19)	0.876 (2)	0.015 (7)*	

### Atomic displacement parameters (Å^2^)

**Table:**

	*U*^11^	*U*^22^	*U*^33^	*U*^12^	*U*^13^	*U*^23^
Re1	0.01570 (6)	0.01934 (6)	0.02291 (6)	−0.00064 (3)	0.01024 (4)	0.00179 (4)
Br1	0.04358 (17)	0.02932 (15)	0.02861 (14)	−0.00256 (12)	0.02177 (13)	−0.00259 (12)
P1	0.0162 (3)	0.0179 (3)	0.0219 (3)	−0.0005 (2)	0.0105 (2)	0.0015 (3)
O1	0.0244 (9)	0.0195 (9)	0.0297 (10)	0.0009 (7)	0.0155 (8)	0.0024 (8)
O2	0.0290 (11)	0.0302 (11)	0.0431 (13)	−0.0090 (9)	0.0139 (10)	0.0058 (10)
O3	0.0185 (10)	0.0465 (14)	0.0523 (14)	0.0029 (9)	0.0110 (10)	0.0147 (11)
O4	0.0445 (13)	0.0394 (13)	0.0460 (13)	−0.0008 (10)	0.0342 (11)	−0.0043 (10)
C1	0.0184 (12)	0.0227 (13)	0.0240 (13)	−0.0042 (10)	0.0122 (10)	−0.0025 (10)
C2	0.0242 (14)	0.0278 (15)	0.0385 (16)	−0.0006 (11)	0.0194 (12)	0.0019 (12)
C3	0.0220 (14)	0.0438 (18)	0.0406 (17)	−0.0017 (12)	0.0201 (13)	−0.0037 (14)
C4	0.0296 (15)	0.0386 (17)	0.0346 (15)	−0.0143 (13)	0.0225 (13)	−0.0074 (13)
C5	0.0296 (14)	0.0285 (14)	0.0261 (13)	−0.0083 (11)	0.0172 (11)	−0.0033 (11)
C6	0.0238 (12)	0.0200 (13)	0.0227 (12)	−0.0051 (10)	0.0136 (10)	−0.0037 (10)
C7	0.0265 (13)	0.0186 (13)	0.0242 (13)	−0.0033 (10)	0.0147 (11)	−0.0013 (10)
C8	0.0219 (13)	0.0190 (13)	0.0375 (15)	0.0028 (10)	0.0151 (12)	0.0069 (11)
C9	0.0440 (19)	0.0245 (16)	0.054 (2)	0.0056 (13)	0.0251 (17)	−0.0042 (14)
C10	0.0322 (16)	0.0329 (17)	0.0437 (19)	−0.0016 (12)	0.0171 (14)	0.0165 (14)
C11	0.0243 (13)	0.0342 (15)	0.0253 (14)	0.0002 (11)	0.0127 (11)	−0.0040 (12)
C12	0.0294 (16)	0.053 (2)	0.0275 (16)	−0.0033 (14)	0.0055 (13)	−0.0007 (14)
C13	0.0361 (16)	0.0339 (17)	0.0338 (16)	−0.0083 (13)	0.0184 (13)	−0.0114 (13)
C14	0.0182 (12)	0.0294 (15)	0.0268 (14)	0.0000 (10)	0.0106 (10)	0.0037 (11)
C15	0.0241 (14)	0.0283 (15)	0.0338 (15)	−0.0038 (11)	0.0152 (12)	0.0044 (12)
C16	0.0210 (12)	0.0241 (14)	0.0316 (14)	0.0002 (10)	0.0151 (11)	0.0033 (11)

### Geometric parameters (Å, º)

**Table:**

Re1—C14	1.901 (3)	C5—H5	0.9300
Re1—C15	1.943 (3)	C6—C7	1.468 (4)
Re1—C16	1.915 (3)	C7—H7	1.01 (3)
Re1—O1	2.1739 (18)	C8—C9	1.514 (5)
Re1—P1	2.4655 (13)	C8—C10	1.528 (4)
Re1—Br1	2.6116 (6)	C8—H8	0.9800
P1—C1	1.835 (3)	C9—H9A	0.9600
P1—C8	1.842 (3)	C9—H9B	0.9600
P1—C11	1.865 (3)	C9—H9C	0.9600
O1—C7	1.231 (3)	C10—H10A	0.9600
O2—C14	1.153 (3)	C10—H10B	0.9600
O3—C15	1.144 (3)	C10—H10C	0.9600
O4—C16	1.148 (3)	C11—C13	1.517 (4)
C1—C2	1.392 (4)	C11—C12	1.533 (4)
C1—C6	1.406 (4)	C11—H11	0.9800
C2—C3	1.393 (4)	C12—H12A	0.9600
C2—H2	0.9300	C12—H12B	0.9600
C3—C4	1.371 (5)	C12—H12C	0.9600
C3—H3	0.9300	C13—H13A	0.9600
C4—C5	1.383 (4)	C13—H13B	0.9600
C4—H4	0.9300	C13—H13C	0.9600
C5—C6	1.405 (3)		
			
C14—Re1—C16	90.87 (12)	O1—C7—H7	118.4 (15)
C14—Re1—C15	89.06 (12)	C6—C7—H7	112.5 (15)
C16—Re1—C15	89.00 (12)	C9—C8—C10	111.4 (3)
C14—Re1—O1	174.15 (10)	C9—C8—P1	112.9 (2)
C16—Re1—O1	94.97 (9)	C10—C8—P1	109.7 (2)
C15—Re1—O1	91.35 (10)	C9—C8—H8	107.5
C14—Re1—P1	96.71 (8)	C10—C8—H8	107.5
C16—Re1—P1	91.28 (8)	P1—C8—H8	107.5
C15—Re1—P1	174.22 (8)	C8—C9—H9A	109.5
O1—Re1—P1	82.87 (5)	C8—C9—H9B	109.5
C14—Re1—Br1	91.10 (9)	H9A—C9—H9B	109.5
C16—Re1—Br1	177.00 (8)	C8—C9—H9C	109.5
C15—Re1—Br1	88.77 (10)	H9A—C9—H9C	109.5
O1—Re1—Br1	83.07 (5)	H9B—C9—H9C	109.5
P1—Re1—Br1	90.74 (3)	C8—C10—H10A	109.5
C1—P1—C8	104.98 (12)	C8—C10—H10B	109.5
C1—P1—C11	102.31 (13)	H10A—C10—H10B	109.5
C8—P1—C11	104.94 (14)	C8—C10—H10C	109.5
C1—P1—Re1	111.71 (9)	H10A—C10—H10C	109.5
C8—P1—Re1	118.89 (9)	H10B—C10—H10C	109.5
C11—P1—Re1	112.42 (10)	C13—C11—C12	110.0 (3)
C7—O1—Re1	136.60 (18)	C13—C11—P1	111.6 (2)
C2—C1—C6	117.4 (2)	C12—C11—P1	113.6 (2)
C2—C1—P1	120.9 (2)	C13—C11—H11	107.1
C6—C1—P1	121.56 (19)	C12—C11—H11	107.1
C1—C2—C3	121.7 (3)	P1—C11—H11	107.1
C1—C2—H2	119.1	C11—C12—H12A	109.5
C3—C2—H2	119.1	C11—C12—H12B	109.5
C4—C3—C2	120.4 (3)	H12A—C12—H12B	109.5
C4—C3—H3	119.8	C11—C12—H12C	109.5
C2—C3—H3	119.8	H12A—C12—H12C	109.5
C3—C4—C5	119.6 (3)	H12B—C12—H12C	109.5
C3—C4—H4	120.2	C11—C13—H13A	109.5
C5—C4—H4	120.2	C11—C13—H13B	109.5
C4—C5—C6	120.5 (3)	H13A—C13—H13B	109.5
C4—C5—H5	119.7	C11—C13—H13C	109.5
C6—C5—H5	119.7	H13A—C13—H13C	109.5
C5—C6—C1	120.4 (2)	H13B—C13—H13C	109.5
C5—C6—C7	113.0 (2)	O2—C14—Re1	177.4 (3)
C1—C6—C7	126.6 (2)	O3—C15—Re1	178.6 (3)
O1—C7—C6	129.1 (2)	O4—C16—Re1	177.2 (2)
